# 4,6-Dichloro-2-[(*E*)-(2-{[(*E*)-3,5-dichloro-2-oxidobenzyl­idene]aza­nium­yl}eth­yl)iminiumylmeth­yl]phenolate

**DOI:** 10.1107/S160053681202870X

**Published:** 2012-06-30

**Authors:** Reza Kia, Hadi Kargar, Amir Adabi Ardakani, Muhammad Nawaz Tahir

**Affiliations:** a Department of Chemistry, Science and Research Branch, Islamic Azad University, Tehran, Iran; b Department of Chemistry, Payame Noor University, PO Box 19395-3697 Tehran, Iran; c Ardakan Branch, Islamic Azad University, Ardakan, Iran; d Department of Physics, University of Sargodha, Punjab, Pakistan

## Abstract

The asymmetric unit of the title compound, C_16_H_12_Cl_4_N_2_O_2_, comprises half of a potentially tetra­dentate Schiff base ligand, located about a twofold rotation axis which bis­ects the central C—C bond of the ethane-1,2-diamine group. In the solid state, the compound exists in the zwitterionic form. There are two intra­molecular N—H⋯O hydrogen bonds making *S*(6) ring motifs. In the crystal, mol­ecules are linked by C—H⋯O hydrogen bonds, forming two-dimensional frameworks which lie parallel to (100). There are also short Cl⋯Cl [3.4395 (9) Å] contacts present.

## Related literature

For standard bond lengths, see: Allen *et al.* (1987 ▶). For hydrogen-bond motifs, see: Bernstein *et al.* (1995 ▶). For van der Waals radii, see: Bondi (1964 ▶). For related Schiff base ligands, see: Kargar *et al.* (2011 ▶); Kia *et al.* (2010 ▶).
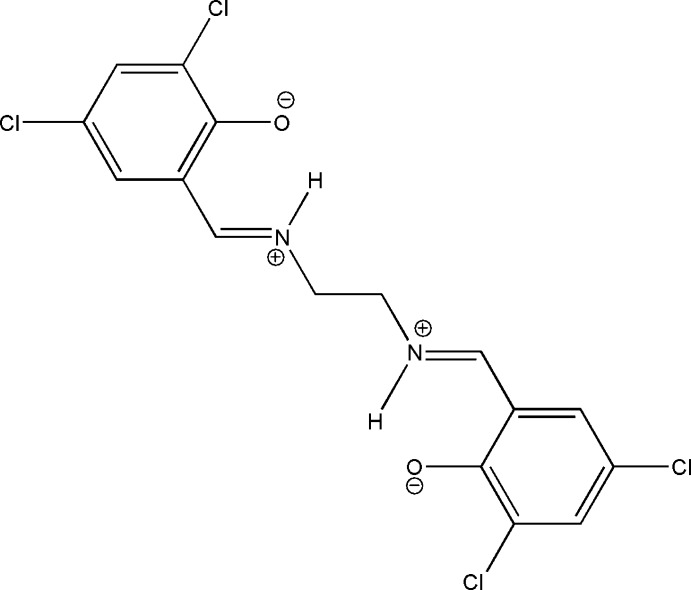


## Experimental

### 

#### Crystal data

C_16_H_12_Cl_4_N_2_O_2_
*M* *_r_* = 406.08
Monoclinic, 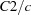

*a* = 20.0505 (14) Å
*b* = 10.1460 (9) Å
*c* = 9.0579 (6) Å
β = 114.955 (4)°
*V* = 1670.6 (2) Å^3^
*Z* = 4
Mo *K*α radiation
μ = 0.72 mm^−1^
*T* = 291 K
0.21 × 0.14 × 0.10 mm

#### Data collection

Bruker SMART APEXII CCD area-detector diffractometer
Absorption correction: multi-scan (*SADABS*; Bruker, 2005 ▶) *T*_min_ = 0.864, *T*_max_ = 0.932
6453 measured reflections
1838 independent reflections
1146 reflections with *I* > 2σ(*I*)
*R*_int_ = 0.040

#### Refinement

*R*[*F* ^2^ > 2σ(*F* ^2^)] = 0.042
*wR*(*F* ^2^) = 0.097
*S* = 0.97
1838 reflections
109 parameters
H-atom parameters constrained
Δρ_max_ = 0.24 e Å^−3^
Δρ_min_ = −0.28 e Å^−3^

### 

Data collection: *APEX2* (Bruker, 2005 ▶); cell refinement: *SAINT* (Bruker, 2005 ▶); data reduction: *SAINT*; program(s) used to solve structure: *SHELXS97* (Sheldrick, 2008 ▶); program(s) used to refine structure: *SHELXL97* (Sheldrick, 2008 ▶); molecular graphics: *SHELXTL* (Sheldrick, 2008 ▶); software used to prepare material for publication: *SHELXTL* and *PLATON* (Spek, 2009 ▶).

## Supplementary Material

Crystal structure: contains datablock(s) global, I. DOI: 10.1107/S160053681202870X/su2459sup1.cif

Structure factors: contains datablock(s) I. DOI: 10.1107/S160053681202870X/su2459Isup2.hkl

Additional supplementary materials:  crystallographic information; 3D view; checkCIF report

## Figures and Tables

**Table 1:** Hydrogen-bond geometry (Å, °)

*D*—H⋯*A*	*D*—H	H⋯*A*	*D*⋯*A*	*D*—H⋯*A*
N1—H1⋯O1	0.97	1.74	2.585 (3)	143
C8—H8*A*⋯O1^i^	0.97	2.55	3.436 (3)	152

Symmetry code: (i) 
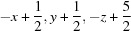
.

**Fig. 1.**
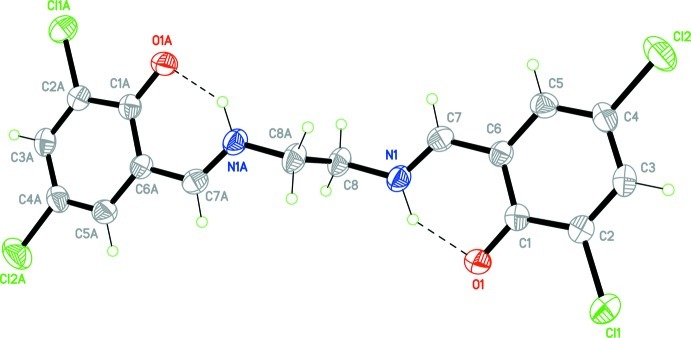
The molecular structure of the title compound, showing 40% probability displacement ellipsoids and the atomic numbering [symmetry code for suffix A = -x + 1/2, -y + 3/2, -z + 2].

**Fig. 2.**
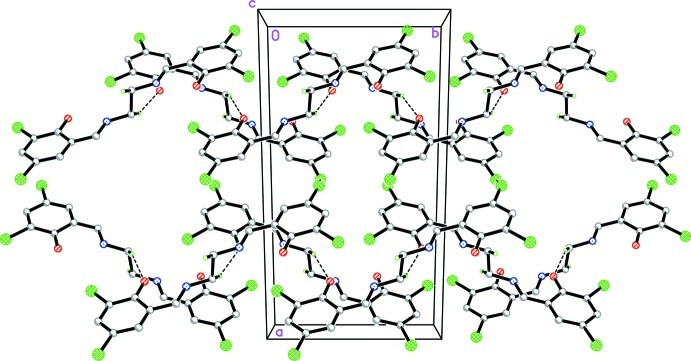
The crystal packing of the title compound viewed down the c-axis, showing hoe the molecules are linked via C—H···O interactions (dashed lines), so forming two dimensional networks (see Table 1 for details).

**Fig. 3.**
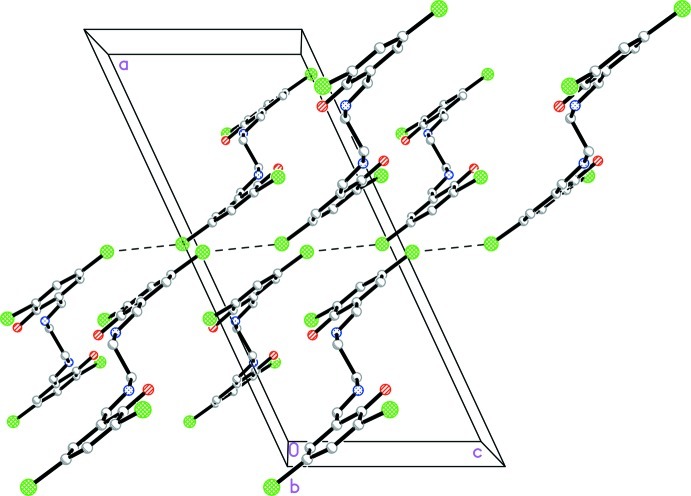
The crystal packing diagram of the title compound viewed down the b-axis, showing the short intermolecular Cl···Cl contacts (dashed lines). All H atoms have been omitted for clarity.

## supplementary crystallographic information

### Comment

In continuation of our work on the crystal structure of Schiff base ligands (Kargar *et al.*, (2011); Kia *et al.*, (2010), we report herein on the crystal structure of the title compound.

The asymmetric unit of the title compound, Fig. 1, comprises half of a potentially tetradentate Schiff base ligand that exists in the keto-amine tautomeric form. The molecule is located about a two-fold rotation axis which bisects the central C8-C8a bond of the ethane-1,2-diamine group. The bond lengths (Allen *et al.*, 1987) and angles are within the normal ranges. The intramolecular N—H···O hydrogen bonds make *S(6)* ring motifs (Table 1; Bernstein *et al.*, 1995).

In the crystal, molecules are linked by C—H···O hydrogen bonds along the *b* and *c* axes directions, forming two dimensional networks which lie parallel to the bc plane [Table 1 and Fig. 2]. There are also short Cl···Cl^ii^ [3.4384 (10)Å; symmetry code: (ii) -x, y, 1/2 - z] contacts present, which are shorter than the sum of the van der waals radius of Cl atoms (Bondi, 1964; Fig. 3).

### Experimental

The title compound was synthesized by adding 3,5-dichlorosalicylaldehyde (2 mmol) to a solution of ethylenediamine (1 mmol) in ethanol (30 ml). The mixture was refluxed with stirring for 30 min. The resultant solution was filtered. Yellow single crystals of the title compound, suitable for *X*-ray structure determination, were obtained by recrystallization from ethanol by slow evaporation of the solvents at room temperature over several days.

### Refinement

The N-bound H atom was located in a difference Fourier map. It was constrained to ride on the parent N-atom with U_iso_(H) = 1.2 U_eq_(N). The C-bound H atoms were included in calculated positions and treated as riding atoms: C—H = 0.93 and 0.97 Å for CH and CH_2_ H atoms, respectively, with U_iso_(H) = 1.2U_eq_(C).

### Crystal data

**Table:**

C_16_H_12_Cl_4_N_2_O_2_	*F*(000) = 824
*M**_r_* = 406.08	*D*_x_ = 1.614 Mg m^−^^3^
Monoclinic, *C*2/*c*	Mo *K*α radiation, λ = 0.71073 Å
Hall symbol: -C 2yc	Cell parameters from 936 reflections
*a* = 20.0505 (14) Å	θ = 2.5–27.5°
*b* = 10.1460 (9) Å	µ = 0.72 mm^−^^1^
*c* = 9.0579 (6) Å	*T* = 291 K
β = 114.955 (4)°	Block, yellow
*V* = 1670.6 (2) Å^3^	0.21 × 0.14 × 0.10 mm
*Z* = 4	

### Data collection

**Table:**

Bruker SMART APEXII CCD area-detector diffractometer	1838 independent reflections
Radiation source: fine-focus sealed tube	1146 reflections with *I* > 2σ(*I*)
Graphite monochromator	*R*_int_ = 0.040
φ and ω scans	θ_max_ = 27.1°, θ_min_ = 2.2°
Absorption correction: multi-scan (*SADABS*; Bruker, 2005)	*h* = −25→25
*T*_min_ = 0.864, *T*_max_ = 0.932	*k* = −7→12
6453 measured reflections	*l* = −9→11

### Refinement

**Table:**

Refinement on *F*^2^	Primary atom site location: structure-invariant direct methods
Least-squares matrix: full	Secondary atom site location: difference Fourier map
*R*[*F*^2^ > 2σ(*F*^2^)] = 0.042	Hydrogen site location: inferred from neighbouring sites
*wR*(*F*^2^) = 0.097	H-atom parameters constrained
*S* = 0.97	*w* = 1/[σ^2^(*F*_o_^2^) + (0.0431*P*)^2^] where *P* = (*F*_o_^2^ + 2*F*_c_^2^)/3
1838 reflections	(Δ/σ)_max_ = 0.001
109 parameters	Δρ_max_ = 0.24 e Å^−^^3^
0 restraints	Δρ_min_ = −0.28 e Å^−^^3^

### Special details

**Table:**

Geometry. All esds (except the esd in the dihedral angle between two l.s. planes) are estimated using the full covariance matrix. The cell esds are taken into account individually in the estimation of esds in distances, angles and torsion angles; correlations between esds in cell parameters are only used when they are defined by crystal symmetry. An approximate (isotropic) treatment of cell esds is used for estimating esds involving l.s. planes.
Refinement. Refinement of F^2^ against ALL reflections. The weighted R-factor wR and goodness of fit S are based on F^2^, conventional R-factors R are based on F, with F set to zero for negative F^2^. The threshold expression of F^2^ > 2sigma(F^2^) is used only for calculating R-factors(gt) etc. and is not relevant to the choice of reflections for refinement. R-factors based on F^2^ are statistically about twice as large as those based on F, and R- factors based on ALL data will be even larger.

### Fractional atomic coordinates and isotropic or equivalent isotropic displacement parameters (Å^2^)

**Table:**

	*x*	*y*	*z*	*U*_iso_*/*U*_eq_	
C1	0.15792 (12)	0.3326 (2)	0.9612 (3)	0.0382 (6)	
C2	0.13797 (12)	0.1976 (3)	0.9247 (3)	0.0402 (6)	
C3	0.09283 (12)	0.1562 (3)	0.7713 (3)	0.0451 (6)	
H3	0.0814	0.0672	0.7511	0.054*	
C4	0.06374 (12)	0.2481 (3)	0.6446 (3)	0.0456 (7)	
C5	0.07928 (12)	0.3784 (3)	0.6728 (3)	0.0444 (6)	
H5	0.0591	0.4384	0.5877	0.053*	
C6	0.12553 (12)	0.4231 (2)	0.8292 (3)	0.0376 (6)	
C7	0.14430 (12)	0.5599 (3)	0.8529 (3)	0.0413 (6)	
H7	0.1217	0.6175	0.7661	0.050*	
Cl1	0.17398 (4)	0.08447 (7)	1.08141 (8)	0.0568 (2)	
Cl2	0.00981 (4)	0.19341 (9)	0.44794 (8)	0.0718 (3)	
N1	0.19105 (10)	0.6061 (2)	0.9888 (2)	0.0432 (5)	
H1	0.2100	0.5376	1.0712	0.052*	
C8	0.21355 (13)	0.7436 (3)	1.0075 (3)	0.0479 (6)	
H8A	0.2193	0.7756	1.1130	0.057*	
H8B	0.1761	0.7963	0.9242	0.057*	
O1	0.20257 (9)	0.37045 (17)	1.10541 (17)	0.0482 (5)	

### Atomic displacement parameters (Å^2^)

**Table:**

	*U*^11^	*U*^22^	*U*^33^	*U*^12^	*U*^13^	*U*^23^
C1	0.0382 (12)	0.0382 (16)	0.0418 (14)	−0.0014 (11)	0.0203 (11)	−0.0028 (12)
C2	0.0407 (12)	0.0379 (16)	0.0450 (13)	0.0008 (11)	0.0210 (11)	0.0003 (12)
C3	0.0474 (14)	0.0370 (16)	0.0545 (16)	−0.0089 (12)	0.0250 (13)	−0.0078 (13)
C4	0.0430 (14)	0.0510 (19)	0.0432 (15)	−0.0101 (12)	0.0185 (12)	−0.0109 (13)
C5	0.0411 (13)	0.0533 (19)	0.0395 (13)	−0.0013 (12)	0.0176 (11)	0.0036 (13)
C6	0.0385 (12)	0.0374 (16)	0.0408 (13)	−0.0017 (11)	0.0206 (11)	0.0008 (12)
C7	0.0443 (14)	0.0415 (17)	0.0424 (13)	0.0029 (11)	0.0224 (12)	0.0030 (12)
Cl1	0.0654 (4)	0.0412 (4)	0.0622 (4)	0.0037 (3)	0.0253 (4)	0.0099 (3)
Cl2	0.0669 (5)	0.0858 (7)	0.0498 (4)	−0.0168 (4)	0.0119 (4)	−0.0205 (4)
N1	0.0539 (12)	0.0323 (14)	0.0452 (12)	−0.0036 (10)	0.0227 (10)	0.0003 (10)
C8	0.0610 (15)	0.0300 (16)	0.0576 (16)	−0.0066 (12)	0.0298 (14)	−0.0038 (12)
O1	0.0571 (10)	0.0443 (12)	0.0361 (9)	−0.0042 (9)	0.0128 (8)	0.0005 (8)

### Geometric parameters (Å, º)

**Table:**

C1—O1	1.292 (2)	C5—H5	0.9300
C1—C2	1.426 (3)	C6—C7	1.430 (3)
C1—C6	1.429 (3)	C7—N1	1.281 (3)
C2—C3	1.366 (3)	C7—H7	0.9300
C2—Cl1	1.729 (2)	N1—C8	1.453 (3)
C3—C4	1.401 (3)	N1—H1	0.9724
C3—H3	0.9300	C8—C8^i^	1.529 (5)
C4—C5	1.358 (4)	C8—H8A	0.9700
C4—Cl2	1.740 (2)	C8—H8B	0.9700
C5—C6	1.401 (3)		
			
O1—C1—C2	121.8 (2)	C5—C6—C1	120.7 (2)
O1—C1—C6	122.1 (2)	C5—C6—C7	119.1 (2)
C2—C1—C6	116.1 (2)	C1—C6—C7	120.0 (2)
C3—C2—C1	122.2 (2)	N1—C7—C6	122.7 (2)
C3—C2—Cl1	119.9 (2)	N1—C7—H7	118.7
C1—C2—Cl1	117.91 (18)	C6—C7—H7	118.7
C2—C3—C4	119.8 (2)	C7—N1—C8	121.9 (2)
C2—C3—H3	120.1	C7—N1—H1	111.5
C4—C3—H3	120.1	C8—N1—H1	126.5
C5—C4—C3	120.8 (2)	N1—C8—C8^i^	109.5 (3)
C5—C4—Cl2	119.8 (2)	N1—C8—H8A	109.8
C3—C4—Cl2	119.4 (2)	C8^i^—C8—H8A	109.8
C4—C5—C6	120.5 (2)	N1—C8—H8B	109.8
C4—C5—H5	119.8	C8^i^—C8—H8B	109.8
C6—C5—H5	119.8	H8A—C8—H8B	108.2
			
O1—C1—C2—C3	−177.7 (2)	C4—C5—C6—C1	0.8 (3)
C6—C1—C2—C3	2.4 (3)	C4—C5—C6—C7	176.3 (2)
O1—C1—C2—Cl1	1.4 (3)	O1—C1—C6—C5	177.77 (19)
C6—C1—C2—Cl1	−178.53 (15)	C2—C1—C6—C5	−2.3 (3)
C1—C2—C3—C4	−1.0 (3)	O1—C1—C6—C7	2.3 (3)
Cl1—C2—C3—C4	180.00 (17)	C2—C1—C6—C7	−177.81 (19)
C2—C3—C4—C5	−0.7 (3)	C5—C6—C7—N1	−173.9 (2)
C2—C3—C4—Cl2	177.00 (17)	C1—C6—C7—N1	1.6 (3)
C3—C4—C5—C6	0.8 (3)	C6—C7—N1—C8	175.4 (2)
Cl2—C4—C5—C6	−176.92 (17)	C7—N1—C8—C8^i^	−97.9 (3)

Symmetry code: (i) −*x*+1/2, −*y*+3/2, −*z*+2.

### Hydrogen-bond geometry (Å, º)

**Table:**

*D*—H···*A*	*D*—H	H···*A*	*D*···*A*	*D*—H···*A*
N1—H1···O1	0.97	1.74	2.585 (3)	143
C8—H8*A*···O1^ii^	0.97	2.55	3.436 (3)	152

Symmetry code: (ii) −*x*+1/2, *y*+1/2, −*z*+5/2.
